# The complete chloroplast genome sequence of *Garcinia pedunculata*

**DOI:** 10.1080/23802359.2019.1699470

**Published:** 2019-12-12

**Authors:** Dejun Yang, Qiong Qiu, Linhong Xu, Yumei Xu, Yi Wang

**Affiliations:** a Institute of Tropical Forestry, Yunnan Academy of Forestry, Puwen, People's Republic of China;; b Laboratory of Forest Plant Cultivation and Utilization, Yunnan Academy of Forestry, Kunming, People's Republic of China

**Keywords:** *Garcinia pedunculata*, chloroplast, Illumina sequencing, phylogenetic analysis

## Abstract

The first complete chloroplast genome (cpDNA) sequence of *Garcinia pedunculata* was determined from Illumina HiSeq pair-end sequencing data in this study. The cpDNA is 157,688 bp in length, contains a large single copy region (LSC) of 85,994 bp and a small single copy region (SSC) of 17,656 bp, which were separated by a pair of inverted repeats (IR) regions of 27,017 bp. The genome contains 130 genes, including 85 protein-coding genes, 8 ribosomal RNA genes, and 37 transfer RNA genes. The overall GC content of the whole genome is 36.2%, and the corresponding values of the LSC, SSC, and IR regions are 33.6%, 30.2%, and 42.2%, respectively. Further phylogenomic analysis showed that *G. pedunculata* and *Garcinia mangostana* clustered in a clade in order Malpighiales.

*Garcinia pedunculata* is the species of the genus *Garcinia* within the family Guttiferae. It is the endangered and rare plant in China due to the scarcity. It distributed in Bangladesh, Yunnan and Tibet of China (Li et al. 2017). Their fruits contain a lot of hydroxy citric acid, which can inhibit the synthesis of fat and achieve the effect of weight loss (Sarma et al. 2016). And the fruit of *G. pedunculata* also has the antihyperglycemic, antidiabetic, and antioxidant bioactive (Ali et al. 2017). Therefore, *G. pedunculata* has huge potential medicinal value (Mundugaru et al. 2014). However, there has been no genomic studies on *G. pedunculata*.

Herein, we reported and characterized the complete *G. pedunculata* genome (MN106251). One *G. pedunculata* individual (specimen number: 201807056) was collected from Puwen, Yunnan Province of China (23°31′51″ N, 101°37′42″ E). The specimen is stored at Yunnan Academy of Forestry Herbarium, Kunming, China and the accession number is YAFH0012758. DNA was extracted from its fresh leaves using DNA Plantzol Reagent (Invitrogen, Carlsbad, CA, USA).

Paired-end reads were sequenced by using Illumina HiSeq system (Illumina, San Diego, CA). In total, about 25.6 million high-quality clean reads were generated with adaptors trimmed. Aligning, assembly, and annotation were conducted by CLC de novo assembler (CLC Bio, Aarhus, Denmark), BLAST, GeSeq (Tillich et al. 2017), and GENEIOUS v 11.0.5 (Biomatters Ltd, Auckland, New Zealand). To confirm the phylogenetic position of *G. pedunculata*, other fourteen species of order Malpighiales from NCBI were aligned using MAFFT v.7 (Katoh and Standley 2013). The Auto algorithm in the MAFFT alignment software was used to align the fifteen complete genome sequences and the G-INS-i algorithm was used to align the partial complex sequences. The maximum likelihood (ML) bootstrap analysis was conducted using RAxML (Stamatakis 2006); bootstrap probability values were calculated from 1000 replicates. *Quercus dentata* (MG967555) and *Quercus baronii* (KT963087) were served as the out-group.

The complete *G. pedunculata* plastid genome is a circular DNA molecule with the length of 157,688 bp, contains a large single copy region (LSC) of 85,994 bp and a small single copy region (SSC) of 17,656 bp, which were separated by a pair of inverted repeats (IR) regions of 27,017 bp. The overall GC content of the whole genome is 36.2%, and the corresponding values of the LSC, SSC, and IR regions are 33.6%, 30.2%, and 42.2%, respectively. The plastid genome contained 130 genes, including 85 protein-coding genes, 8 ribosomal RNA genes, and 37 transfer RNA genes. Phylogenetic analysis showed that *G. pedunculata* and *Garcinia mangostana* clustered in a clade in order Malpighiales (Figure 1). The determination of the complete plastid genome sequences provided new molecular data to illuminate the order *Malpighiales* evolution.

**Figure 1.**
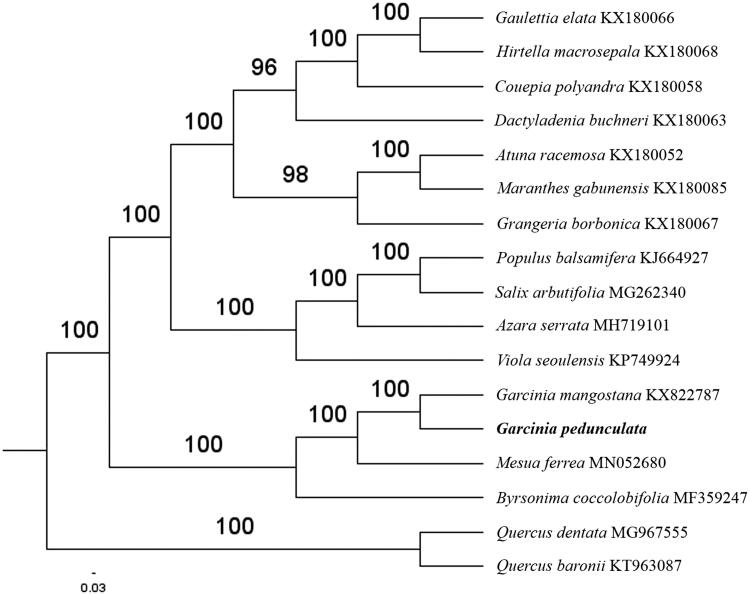
The maximum-likelihood tree based on the 15 chloroplast genomes of *order Malpighiales*. The bootstrap value based on 1000 replicates is shown on each node.
